# Translatability of life‐extending pharmacological treatments between different species

**DOI:** 10.1111/acel.14208

**Published:** 2024-05-26

**Authors:** Daiana Burdusel, Cristin Coman, Diana–Larisa Ancuta, Dirk M. Hermann, Thorsten R. Doeppner, Andrei Gresita, Aurel Popa‐Wagner

**Affiliations:** ^1^ Doctoral School University of Medicine and Pharmacy of Craiova Craiova Romania; ^2^ Chair of Vascular Neurology and Dementia, Department of Neurology University Hospital Essen Essen Germany; ^3^ Cantacuzino National Medical Military Institute for Research and Development Bucharest Romania; ^4^ Department of Neurology University Medical Center Göttingen Göttingen Germany; ^5^ Department of Neurology University of Giessen Medical School Giessen Germany; ^6^ Department of Biomedical Sciences New York Institute of Technology, College of Osteopathic Medicine Old Westbury New York USA

**Keywords:** aging, drug treatments, humans, invertebrates, lifespan, translatability, vertebrates

## Abstract

Anti‐aging research has made significant strides in identifying treatments capable of extending lifespan across a range of organisms, from simple invertebrates to mammals. This review showcases the current state of anti‐aging interventions, highlighting the lifespan extensions observed in animal models through various treatments and the challenges encountered in translating these findings to humans. Despite promising results in lower organisms, the translation of anti‐aging treatments to human applications presents a considerable challenge. This discrepancy can be attributed to the increasing complexity of biological systems, species‐specific metabolic and genetic differences, and the redundancy of metabolic pathways linked to longevity. Our review focuses on analyzing these challenges, offering insights into the efficacy of anti‐aging mechanisms across species and identifying key barriers to their translation into human treatments. By synthesizing current knowledge and identifying gaps in translatability, this review aims to underscore the importance of advancing these therapies for human benefit. Bridging this gap is essential to assess the potential of such treatments in extending the human healthspan.

## Graphical abstract

Despite promising results in lower organisms, translating anti‐aging treatments to human applications presents a considerable challenge. The effectiveness or significance of a targeted molecule from a pathway might differ across species‐specific metabolic scenarios. The importance of targeted signaling pathways varies with the general metabolism of a species. Species of increasing complexity also exhibit higher redundancy in pathways controlling metabolism. The increasing complexity of species reminds us that, much like many aspects of biology and medicine, there exists a law of diminishing returns.

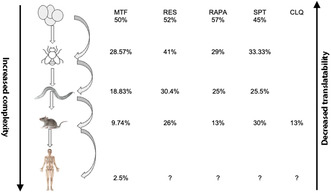

**Abbreviations**
CQ: chloroquine
CR: caloric restriction
CSS: cancer specific survival
DM: *Drosophila melanogaster*
DTT: DJ651‐driven tetanus toxin
EOD: every other day
FFQs: food‐frequency questionnaires
gk99: wrn‐1 strain
HC: high calorie
HCR: high calorie resveratrol
HR: hazard ratio
HS‐LP: high sugar‐low protein
IIS: insulin/IGF‐1 pathway
IRS‐1: insulin receptor substrate
ITP: Intervention Testing Program
LS‐HP: low sugar‐high protein
MD: muscular dystrophy
MLS: maximum lifespan
mLS: medium lifespan
MTF: metformin
mTOR: mamalian target of the rapamycin
ORES: oxyresveratrol
OS: overall survival
OXPHOS: oxidative phosphorylation
P30‐P60: postnatal day 30 to day 60
P4‐P30: postnatal day 4 to day 30
ppm: parts per million
RAPA: rapamycin
RES: resveratrol
RNAi: bec‐1 strain
ROS: reactive oxygen species
SD: standard diet
SodRNAi: Sod1 knockdown
STR: survival time ratio
TJL: The Jackson Laboratory
Trap1: tumor necrosis factor receptor‐associated protein 1
TSC1: tuberous sclerosis protein 1
UM: University of Michigan
UT: University of Texas Health Science Center

## 1 INTRODUCTION

Aging is a complex biological process influenced by many factors, including genetics, lifestyle, and environmental conditions (Mak et al., 2022; Mather, 2022). Understanding the underlying mechanisms interventions to extend lifespan in aging organisms has been the focus of many studies being of interest to both scientists and the general public.

The rapid increase in life expectancy since the 20th century suggests that aging is not solely driven by genetics. Long‐lived individuals without health problems are clustered in certain populations in Greece and Japan, indicating that environment and lifestyle play a significant role. Factors such as diet, education, physical activity, and early life experience all influence mortality (Amorim et al., 2022).

The hallmarks of mammalian aging include genetic instability, telomere shortening, epigenetic alterations, and metabolic disruptions. These disruptions encompass deregulated proteostasis, altered nutrient signaling, and energy shortages due to mitochondrial dysfunction. Together, they reveal a strong connection between aging, age‐related diseases, and the balance of cellular energy. Indeed, accumulating data shows that aging and diseases associated with aging are closely linked to an imbalance between energy supply and demand (Amorim et al., 2022). This suggests that interventions such as exercise, dietary adjustments, and molecules targeting longevity pathways could address these factors (Amorim et al., 2022; Johnson et al., 2013; López‐Otín et al., 2013). Thus, exercise is widely recognized as a crucial lifestyle change that wards off metabolic issues, including obesity and type 2 diabetes mellitus, while a lack of physical activity and a sedentary lifestyle plays a significant role in their development.

Limiting calorie intake is also a well‐established method for preventing metabolic problems associated with aging across various species. When calories are restricted, there is an increase in oxidative phosphorylation (OXPHOS) and antioxidant defenses, as well as the creation of new mitochondria. Furthermore, reducing caloric intake enhances the renewal of mitochondria by promoting the formation of new mitochondria and the removal of damaged ones through the process of mitophagy (Amorim et al., 2022).

Various treatments including rapamycin, resveratrol, spermidine, and others have been proposed as potential lifespan‐extending treatments (Doeppner et al., 2022). However, despite the promising results obtained in organism models such as yeast, worms, and flies, the translatability of these interventions across species of increasing complexity and to humans is still uncertain. Furthermore, the results of lifespan studies in different species and dosages are frequently inconsistent (Hector et al., 2012). Of note, many studies take into account how different types of diets can influence the aging process in distinct ways depending on the organism model (Fontana et al., 2010), adding to the complexity of the field. In addition, the testing of life‐extending treatments in humans raises questions about safety, efficacy, and optimal dosage, making it challenging to identify the most promising candidates for further development.

Among the biological theories of aging, mitochondrial dysfunction, and metabolic dysregulation are key factors, impacting cellular energy management and susceptibility to oxidative stress (López‐Otín et al., 2013). Treatments like rapamycin and resveratrol target these processes; rapamycin modulates the mTOR pathway, enhancing autophagy and mitochondrial health, while resveratrol activates sirtuins to improve mitochondrial function and stress resistance (Johnson et al., 2013). These interventions suggest a pathway to lifespan extension by directly addressing the cellular underpinnings of aging.

This review aims to identify the current state of knowledge on treatments and interventions that have been shown to impact the lifespan of different species. We will compare and contrast the results of different studies, such as dosage and species, to learn more about how these findings can be applied to other species and how they could be utilized in future studies involving humans.

## 2 METHODOLOGY

In this study, our main focus was on exploring the existing literature concerning the translatability of life‐extending treatments across various species. Specifically, we examined the effects of metformin, resveratrol, rapamycin, spermidine, and chloroquine treatments on the lifespan of *Saccharomyces cerevisiae*, *Drosophila melanogaster*, *Caenorhabditis elegans*, mice, rats, and humans. To achieve this objective, we conducted a comprehensive search of pertinent scientific literature using two major databases: PubMed and Web of Science. The search terms we employed included “metformin,” “resveratrol,” “rapamycin,” “spermidine,” “chloroquine,” “lifespan,” “longevity,” “species comparison,” “translational research,” and “aging interventions.” To ensure a comprehensive approach, we considered a wide array of both qualitative and quantitative studies, including primary research articles and review papers.

In selecting the drugs for our analysis, we applied stringent criteria based on their widespread study across multiple species and their documented impact on lifespan and aging‐related processes. These five drugs were chosen because they have been extensively tested and reported in the literature for their effects on longevity in at least five of the six species we selected. This selection allows for a comprehensive cross‐species comparison, facilitating a deeper understanding of the translatability of anti‐aging interventions from model organisms to humans. The inclusion of these specific drugs in our study was also predicated on their diverse mechanisms of action, which target key pathways implicated in aging, such as mTOR signaling, sirtuin activation, autophagy, and metabolic regulation. This diversity ensures a broad examination of potential anti‐aging interventions across the evolutionary spectrum, from simple unicellular organisms to complex mammals, providing insights into the fundamental processes of aging and the potential for these interventions to impact human health and longevity.

Additionally, we included governmental and non‐governmental reports, statistical databases, and regulatory frameworks related to longevity research and aging interventions. Our literature search covered studies published up to the date of this review, ensuring the inclusion of the most recent research findings in the field. Given the flexibility that a review article provides, we employed a thematic analysis to synthesize the findings, identifying patterns and common themes across the selected literature.

## 3 RESULTS

### 3.1 Metformin

Metformin (MTF), an indirect mTOR kinase inhibitor, is a widely recognized oral medication commonly used to manage type 2 diabetes by improving insulin sensitivity and reducing blood sugar levels. It belongs to a class of medications known as biguanides. Beyond its primary role in diabetes management, there has been growing interest in exploring metformin's potential effects on lifespan and aging. Some research suggests that metformin might influence cellular and molecular pathways associated with aging, such as reducing inflammation and enhancing mitochondrial function (Du et al., 2022). These effects have led to investigations into whether metformin could have a positive impact on extending lifespan and promoting healthy aging in various organisms, including model organisms like mice (Martin‐Montalvo et al., 2013). While further studies are needed to fully understand metformin's role in longevity and aging in humans (Stevenson‐Hoare et al., 2023), its potential as a tool for promoting healthy aging continues to be an area of active scientific inquiry.

#### 3.1.1 Effects of metformin on the lifespan of *Saccharomyces*

A comprehensive study examining the impact of 25 mM metformin exposure on *Schizosaccharomyces pombe* revealed a notable extension of chronological lifespan when the compound was administered from the outset. Specifically, the inclusion of metformin in a medium containing 3% glucose led to an approximate 50% increase in chronological lifespan compared to the control group. Furthermore, the investigation analyzed the effects of glucose on lifespan. Intriguingly, it was observed that metformin exhibited an even more pronounced impact on chronological lifespan when the yeast cells were cultivated in a glucose‐free environment, resulting in a remarkable 33% increase in lifespan compared to the counterparts cultured in the presence of 3% glucose (Şeylan & Tarhan, 2023).

In another study, the authors used the budding yeast's, *S. cerevisiae*, as a model for studying aging and age‐related diseases. Previous studies lacked quantitative precision in identifying aging factors, prompting the authors to introduce a refined method. They applied this method to study gene deletions' effects on lifespan, ultimately revealing a connection between protein glycosylation and metformin's ability to extend lifespan. This research highlights the power of competitive‐aging and advanced modeling in understanding the mechanisms of drugs like metformin in extending lifespan from 10.7 to 15.5 days (Avelar‐Rivas et al., 2020).

#### 3.1.2 Effects of metformin on the lifespan of *Drosophila melanogaster*

One study showed that metformin has a dose‐dependent effect on the larvae's development when provided on a 4% amylose starch diet. Diets with metformin concentrations ranging from 0.1 to 25 mM did not significantly affect the larval pupariation time compared to diets without metformin. However, when flies' food was supplemented with the highest concentration of metformin (50 mM), half‐pupariation time was extended by 17%.

Previous research suggests that, within the context of a sucrose diet, increasing the concentration of metformin above 10 mM leads to a dose‐dependent reduction in lifespan (Slack et al., 2012). They also tried an experimental medium with 10 mM metformin. They found that at this concentration, metformin prolonged developmental time by 8% on a low‐starch diet (0.25%) and accelerated pupariation by 11% on a high‐starch diet (20%). This indicates that metformin partially counteracted the negative effects of high‐starch concentrations on fly development. The 10 mM metformin supplementation failed to prevent the negative consequences of a starch diet and decreased the lifespan of both male and female flies (Abrat et al., 2018).

To evaluate the impact of metformin on the lifespan of *D. melanogaster* (*DM*) under different conditions, a study was conducted using wild‐type flies (*Canton S*) and *Trap1*‐deficient flies. *Trap1* (tumor necrosis factor receptor‐associated protein 1) encodes a mitochondrial Hsp90 chaperone. The flies were also subjected to severe hypoxia in a specially designed chamber, followed by a reperfusion period of 120 h. *Trap1* deficiency resulted in significantly shorter lifespans under both normoxic and hypoxic conditions and negatively affected activity and negative geotaxis. However, the introduction of metformin appeared to mitigate the negative effects of *Trap1* deficiency on mortality rates after hypoxia, leading to decreased post‐hypoxic mortality rates of 28.57% (Kokott‐Vuong et al., 2021).

Another study investigated the effects of metformin on the lifespan on *DM* when administered at different concentrations (1–100 mM). For both genders, there were no significant differences in survival between flies maintained on 0, 1, 2.5, and 5 mM metformin. However, males maintained on 100 mM metformin had significantly shorter lifespans than flies maintained on 0–50 mM metformin. On the other hand, females maintained on 25, 50, and 100 mM metformin had significantly shorter lifespans than non‐treated controls and flies fed with 0–10 mM metformin (Slack et al., 2012).

#### 3.1.3 Effects of metformin on the lifespan of *Caenorhabditis elegans*

The effect of metformin on lifespan was also tested on *C. elegans* cocultured with *Escherichia coli*. The study found that metformin increases lifespan by altering microbial folate and methionine metabolism. The percentage of median lifespan increase and maximum lifespan varied depending on the *E. coli* strain metformin sensitivity and glucose concentration. They reported an increment of lifespan with 18%, 36%, and 3% for 25, 50, and 100 mM of metformin, respectively (Cabreiro et al., 2013).

Another report showed that the lifespan of *C. elegans* was extended by 15 days compared with 12 days control group, when treated with metformin at young ages (3 days old). However, when treatment was initiated at old age (10 days old), metformin was found to be toxic at all tested doses (10, 25, and 50 mM), and shortened the lifespan, that is, 11 versus 15 days. This indicates an age‐dependent decrease in metformin tolerance, which culminated in late‐life toxicity of all metformin doses tested, indicating possible safety risks of late‐life metformin administration (Espada et al., 2020).

#### 3.1.4 Effects of metformin on the lifespan of mice

In a study conducted on male mice, supplementation with 0.1% (w/w) metformin led to a significant extension of mean lifespan by 5.83% and 4.15% in two different strains of mice, C57BL/6 and B6C3F1. Higher concentrations of metformin (1% w/w) were toxic and significantly shortened mean lifespan by 14.4% in C57BL/6 mice, likely due to renal failure. Male mice treated with 0.1% metformin did not show any major differences in pathologies or obvious causes of death compared to control mice (Martin‐Montalvo et al., 2013).

Another study reported that metformin given in drinking water at a concentration of 0.13 μg/mL significantly increased the medium and maximum lifespan of SHR mice by 37.8% and 10.3%, respectively. Indeed, fibroblasts obtained from the skin of metformin‐treated mice showed a significant delay in the accumulation of markers of cellular senescence compared to control animals (Arkadieva et al., 2011).

#### 3.1.5 Effects of metformin on human health

A 20‐year clinical study assessing the impact of metformin on longevity in individuals with type 2 diabetes and matched non‐diabetic peers revealed that metformin initially conferred better survival within the first 3 years but later led to shorter survival times than controls after 5 years of treatment. The survival time ratio (STR) for metformin‐treated patients was 0.819, indicating 81.9% of the control's survival. Sulphonylurea‐treated patients exhibited even worse survival outcomes (STR of 0.799), while patients on both medications showed decreased survival for metformin patients (STR of 0.693). The hazard ratio (HR) indicated that metformin and sulphonylurea patients had a 69% and 59% higher likelihood of death, respectively, than non‐diabetic controls. To conclude, while metformin demonstrated initial benefits, the study suggests that these were overshadowed by the long‐term effects of type 2 diabetes over two decades (Stevenson‐Hoare et al., 2023).

A comprehensive meta‐analysis, involving 20 studies and 13,008 participants investigated the relationship between metformin and overall survival (OS) as well as cancer‐specific survival (CSS) among individuals with both cancer and type 2 diabetes. The findings illuminated a notable survival advantage associated with metformin usage when compared to alternative glucose‐lowering treatments, evident in both OS and CSS outcomes. Importantly, this favorable survival trend extended across various cancer types and countries.

The study's implications underscored metformin's potential as the preferred therapeutic option for individuals dealing with concurrent type 2 diabetes and cancer, as it exhibited enhanced OS (HR = 0.66) and CSS (HR = 0.62) compared to alternative diabetic medications (Yin et al., 2013).

### 3.2 Resveratrol

Resveratrol (3, 5, and 4′‐trihydroxy‐trans‐stilbene) is a bioflavonoid that is found in a variety of foods, including grapes, berries, and peanuts. It has been the subject of numerous studies examining its potential health benefits, including its effect on lifespan (Bass et al., 2007; Bhullar & Hubbard, 2015). Some studies have shown that resveratrol (RES) can extend the lifespan of certain species, such as yeast, worms, flies, and mice, by activating a group of enzymes called sirtuins. Sirtuins are a family of enzymes that are involved in a variety of biological processes, including metabolism, DNA repair, and regulation of gene expression. One member of this family, SIRT1, has been extensively studied in the context of aging and lifespan extension (Lee et al., 2016; Wang et al., 2013).

#### 3.2.1 Effects of resveratrol on the lifespan of *Saccharomyces cerevisiae*

In yeast, resveratrol exhibits a remarkable ability to replicate the positive effects of calorie restriction on lifespan extension. This is achieved through its capacity to stimulate Sir2, a crucial protein involved in maintaining DNA stability and repair processes within the cell. This activation leads to an extension of the yeast's lifespan, often by as much as 70% (Howitz et al., 2003).

Pannakal et al. demonstrated a dose‐dependent capacity to increase chronological lifespan of *S. cerevisiae*, with the most pronounced effect seen at 1 μg/mL. This concentration significantly extended mean lifespan from 5.61 ± 0.10 days (for the vehicle control) to 7.48 ± 0.07 days (at 10 μg/mL) and 8.44 ± 0.04 days (at 1 μg/mL). At the optimal 1 μg/mL concentration, the yeast extract leads to a substantial 34% increase in mean lifespan and an impressive 41% extension of overall lifespan. While the TORC1 signaling pathway plays a role in this extension, it is noteworthy that the *Gcn5*, *Sir2*, and *Snf1* pathways are not required, suggesting a pathway‐independent mechanism for this lifespan enhancement (Pannakal et al., 2017).

#### 3.2.2 Effects of resveratrol on the lifespan of *Drosophila melanogaster*

In a representative study on *DM*, resveratrol was added to five different diets (CR calorie restriction diet, HS‐LP high sugar–low protein diet, LS‐HP low sugar–high protein diet) at concentrations of 100, 200, or 400 μM, 48 h after hatching, to male and female groups of wild‐type Canton S and sodRNA1 knockdown flies, respectively. Thus, for the Canton S flies kept on the base diet or the calorie‐restricted base diet, no effect on the lifespan was reported for either 100 or 200 μM RES. Male *Sod1* knockdown (sodRNAi) worms kept on a base diet supplemented with 400 μM RES experienced a lifespan *decrease* of 9.6%. However, 200 μM RES increased the lifespan of female *Sod1* knockdown worms (sodRNAi) kept on a standard diet by 8.7%. The change in the survival percentage decreased to 7% at 400 μM RES. The highest significant increase in lifespan, approximately 15%, was observed in association with an LS‐HP diet and 200 μM RES in Canton S females. In this study, 100 μM RES did not influence the lifespan. In contrast, Canton S males kept on the LS‐HP diet experienced a small lifespan increase (7.8%) at 100 μM that decreased to 3% at the 200 μM dose. For the Canton S flies kept on the base diet or the calorie‐restricted base diet, no effect on the lifespan was reported for either 100 or 200 μM RES. Resveratrol had no significant effect on LS when they were kept on an HS‐LP diet. On the contrary, RES had a significant effect on PS females, with an increase of 11.4% in sodRNAi worms kept on the HS‐LP diet (Wang et al., 2013). The different results observed between the wild‐type and *Sod1* knockdowns suggest the potential involvement of the enzyme superoxide dismutase 1 in resveratrol's mechanism of action. This could be due to lower levels of this enzyme in *Sod1* knockdown flies (Denu, 2005; Martin et al., 2009).

Another study examined the effect of RES on *DM*, utilizing male and female flies of the ORR and Harwich strains. The treatment started 48 h after eclosion, and the flies were kept either on cornmeal (control) or corneal supplemented with 31.54 μg/L RES. When compared to the control group, the findings revealed that both male and female flies who received RES had a longer lifespan, with an increase of up to 40.7% in the ORR strain and 41.4% in the Harwich strain with similar results for both genders (Islam et al., 2019).

In a study conducted by Khan et al., the researchers examined the effects of resveratrol‐enriched rice callus (at a concentration of 5.7 mg/L) on 48‐h‐old male and female ORR and Harwich flies, under similar experimental conditions to ensure comparability. The results showed that the resveratrol rice DJ526 callus had a significant impact on the median lifespan of all the wild‐type flies tested—ORR males and females, Harwich males and females. Specifically, it extended their median lifespans by 30%, 20%, 50%, and 36%, respectively, when compared to the control group. Interestingly, notable differences were observed in the extent of the lifespan extension between males and females (Khan et al., 2019).

Another experiment compared the effect of resveratrol supplementation on DTT (DJ651‐driven tetanus toxin) and wild‐type Canton S flies. Significant results were observed at a concentration of 200 μM RES, which increased the lifespan in DTT males by 9% and in females by 8%. In Canton S flies, the lifespan increased by 10% in males and 17% in females (Bauer et al., 2004).

#### 3.2.3 Effects of resveratrol on the lifespan of *Caenorhabditis elegans*

Next, we examined the effect of resveratrol and oxyresveratrol on the lifespan of *C. elegans*. Both RESl and oxyresveratrol (ORES) increased the mean lifespan (MLS) of the wild‐type worms compared to the control group. The MLS of worms treated with RES at concentrations of 100, 500, and 1000 μM increased by 22.2%, 23.7%, and 30.4%, respectively. The MLS of worms treated with ORES at concentrations of 100, 500, and 1000 μM increased by 7.4%, 17.8%, and 31.1%, respectively. These results suggest that both RES and ORES can extend the lifespan of *C. elegans*, with RES having a more substantial effect. However, the sir‐2.1 mutants did not exhibit a lifespan increase. Sir‐2.1 is a member of the Sir‐2 family of NAD+‐dependent protein deacetylases, has been shown to regulate nematode aging via the insulin/IGF pathway transcription factor *daf‐16*. However, the mechanisms behind this lifespan extension and whether it is dependent on the Sir‐2.1 gene were not examined in this study (Lee et al., 2016).

The effects of the antioxidant and radical scavenging activity of resveratrol have been mostly tested in *C. elegans* treated with free radical‐producing chemicals, such as juglone and paraquat. Thus, under stressful conditions, *C. elegans* wild‐type N2 var. Bristol strain pretreated with RES at concentrations of 50, 100, and 200 μM for 72 h at 18°C experienced an expansion of lifespan by 53.1%, 30.1%, and 15.6%, respectively. The lethal stressor used was 10 μg/mL juglone (Rea et al., 2005).

Two‐day‐old *C. elegans* wild‐type strain nematodes were treated with 50 μM of RES to assess its effect on lifespan. The results showed an enhancement of medium lifespan (mLS) and maximum lifespan (MLS) by 64% and 30%, respectively. Additionally, when exposed to highly toxic paraquat, the treated group had a higher average survival time (Gruber et al., 2007).

#### 3.2.4 Effects of resveratrol on the lifespan of mice

In a study focused on understanding the impact of dietary and resveratrol interventions on longevity, cohorts of middle‐aged male C57BL/6NIA mice were strategically divided. These groups were exposed to distinct dietary regimens: a standard diet (SD) and a high‐calorie (HC) diet composed of 60% fat calories. To explore the potential of resveratrol, two varying concentrations were incorporated into both diet categories—approximately 5.2 and 22.4 mg/kg/day. The results revealed among mice on the HC diet, those treated with resveratrol (HCR group) exhibited a distinct survival advantage, a phenomenon that became apparent at around 60 weeks. By the time the study reached 114 weeks, the disparity in survival rates became more pronounced: 58% of mice kept on HC diet had succumbed, whereas a substantially lower 42% mortality rate was observed among both HCR mice and those on the SD (Baur et al., 2006). Additionally, combining the effects of alternate‐day fasting with the lower dose of resveratrol extended both mean and maximum lifespans by 15% in comparison to SD controls. However, applying a higher dose of resveratrol from the age of 12 months (SDHR) did not significantly influence longevity. Notably, under an SD regimen, resveratrol failed to enhance OS or maximum lifespan (Miller et al., 2011; Pearson et al., 2008).

In humans, resveratrol has been tested in an open‐label, single‐arm, phase IIa trial to determine if it provides therapeutic benefits to patients with Duchenne, Becker, or Fukuyama muscular dystrophies. The results suggest that resveratrol offers benefits in terms of quantitative muscle testing and serum creatine kinase levels for these patients (Kawamura et al., 2020).

### 3.3 Rapamycin

Rapamycin (RAPA) is a drug originally discovered in a soil sample from Easter Island. It has been shown to have anti‐aging properties in various organisms including yeast, worms, flies, and mice. The mechanisms by which rapamycin extends lifespan are not yet fully understood, but it is believed to work by inhibiting the target of the rapamycin (TOR) signaling pathway, which plays a role in growth, proliferation, and aging (Villa‐Cuesta et al., 2014). Rapamycin has also been found to improve healthspan, which refers to the period of life during which an organism is healthy and able to function well.

#### 3.3.1 Effects of rapamycin on the lifespan of *Saccharomyces*

A study that investigated the effect of rapamycin on the lifespan of yeast at various concentrations, ranging from 0.1 to 40 nM, revealed that rapamycin extends MLS in yeast strains, except for atg1Δ and atg7Δ strains, which are deficient in autophagy‐related genes. The most significant extension of lifespan occurs at 10, 20, and 40 nM concentrations. Interestingly, lower concentrations of rapamycin (0.1 and 1 nM) do not impact CLS. Notably, the extension of lifespan correlates with rapamycin‐induced upregulation of macroautophagy during chronological aging. These findings emphasize the role of autophagy in rapamycin's ability to extend yeast lifespan, suggesting that autophagy modulation may hold promise for enhancing longevity and healthspan (Alvers et al., 2009).

In another research study that examined the impact of caffeine and rapamycin on chronological lifespan in yeast maintained on two different types of medium, namely, YES media (with 3% glucose) and EMM (with 2% glucose), it was revealed that in the nutrient‐rich medium, rapamycin exerted a notable increase in lifespan compared to caffeine and the control group. When compared to the control group, rapamycin treatment resulted in a 57% increase in lifespan (Rallis et al., 2013).

#### 3.3.2 Effects of rapamycin on the lifespan of *Drosophila melanogaster*

The idea that rapamycin treatment during early developmental stages contributes to longevity control was assessed experimentally using the *wiso31 Drosophila* strain for both males and females. Thus, the treatment was initiated during the third larval stage, employing varied rapamycin concentrations (1, 50, or 200 μM) from 72 h post‐egg laying until either the pupal stage or days 0 to 10, and days 10 to 20. The findings revealed distinct effects of different rapamycin concentrations on lifespan. For concentrations of 1 and 50 μM rapamycin, there was no significant change in median lifespan observed in either males or females, irrespective of the treatment window. In contrast, administering 200 μM rapamycin extended the median lifespan when examining both genders collectively. Specifically, treatment of males during days 0–10 resulted in a significant increase in median lifespan, rising from 61 days (control) to 66 days (rapamycin‐treated). Interestingly, female flies did not show a significant increase in median lifespan during this treatment period. To confirm that the time frame of treatment is more important than the duration of treatment, the researchers additionally administered 200 μM rapamycin from day 10 to 20, yet this had no impact on lifespan. These findings highlight the gender‐specific impact of early‐stage rapamycin treatment on *DM* longevity, reinforcing the importance of precise timing for achieving significant effects (Aiello et al., 2022).

Another study investigated the effect of rapamycin treatment on the lifespan of various strains of *D. melanogaster*, including wild‐type, null mutants, and strains with manipulated gene expression. The results showed that a concentration of 200 μM resulted in a 13% increase in lifespan for female wild‐type flies, while male flies showed a 6% increase. Female flies exposed to lower (50 μM) and higher (400 μM) concentrations exhibited a 9% and 10% increase in lifespan, respectively. Additionally, the study examined the impact of rapamycin treatment under paraquat treatment conditions. The findings revealed that the flies experienced a 70% increase under oxidative stress conditions induced by paraquat injection, suggesting that the effect of rapamycin on lifespan involves more factors than just targeting the nutrient‐sensing TOR pathway, as the enhancement of the stress resistance of the cell through upregulation of antioxidant defense systems or enhancement of DNA repair, mechanisms autophagy activation that can also help the reduction of the reactive oxygen species (ROS) and a metabolic shift toward enhance mitochondrial efficiency or alter energy production pathways to reduce ROS production and reduce oxidative damage (Bjedov et al., 2010; Shaposhnikov et al., 2022).

*DM*'s lifespan is largely determined by amino acid imbalances, and food restriction is typically performed by diluting yeast as a protein source (Grandison et al., 2009). The effect of rapamycin on the lifespan of Drosophila, when fed various yeast diets known to extend longevity, was investigated. The researchers also included a starvation diet consisting of 0.8% w/v agar in water without additional nutrients. Rapamycin was added to the diets at various concentrations (5, 50, 100, 200, and 400 μM) to examine its dose‐dependent effects and the presence‐absence effects on the survival of Drosophila strains wDha and OreR. The study found that rapamycin's impact on *D. melanogaster* (DM) lifespan varied depending on the diet, with some diets showing a significant increase in lifespan and others showing no effect. The most promising results were observed at rapamycin concentrations ranging from 50 to 200 μM, which significantly extended the MLS of flies fed a 2% yeast diet. The maximum extension was achieved at 200 μM rapamycin with a lifespan increment of 9% (Villa‐Cuesta et al., 2014).

#### 3.3.3 Effects of rapamycin on the lifespan of *Caenorhabditis elegans*

Transcriptomic studies were conducted using *C. elegans* (CL2070) to explore the impact of rapamycin treatment on lifespan and the accumulation of insoluble proteins as organisms age. To ensure age synchronization in the study, a germline‐deficient strain (JK1107) was utilized to generate cohorts of worms. At 72 h of life, the worms were administered either 100 μM rapamycin or a control vehicle. The findings revealed that the rapamycin treatment led to a mere 9% increase in lifespan. Furthermore, compared to the control group, the animals receiving rapamycin only achieved this level of extension on day 8 of life (Yee et al., 2021).

Another study showed that treatment of *C. elegans* WT with rapamycin had a maximum lifespan of 31.35 days, which is a 29% increase compared to the control group (Robida‐Stubbs et al., 2012).

#### 3.3.4 Effects of rapamycin on the lifespan of mice

The National Institute on Aging's Intervention Testing Program (ITP) conducted a pioneering study across three locations: The Jackson Laboratory, the University of Michigan (UM), and the University of Texas Health Science Center (UT). The research identified rapamycin as the first drug able to extend the maximum lifespan of mice. In this study, mice were provided with food containing 14 parts per million (ppm) of rapamycin. The investigation involved both male and female mice and initiated treatment either after 270 days or after 600 days of age. The study found that both treatment timelines effectively extended the maximum lifespan of UM‐HET3 mice by 14% for females and 9% for males (Harrison et al., 2009). Furthermore, researchers observed that the combination of rapamycin and acarbose, a drug used for managing type 2 diabetes, substantially extended the median lifespan of 9‐month‐old UM‐HET3 mice. The study revealed an average lifespan increase of 28% for female mice and 34% for male mice. When the same treatment regimen was applied to 16‐month‐old mice, an overall 13% lifespan extension was observed for both genders. A closer look at the data revealed that the treatment consistently improved longevity for females across all labs, while for males, only the UT lab showed a significant 32% lifespan increase (Miller et al., 2011). In a subsequent investigation at the ITP location, it was found that the oral administration of 4.7, 14.4, or 42 ppm of rapamycin to UM‐HET3 mice, initiated at 9 months, had a significant positive effect on the maximum and median lifespan of female mice. Furthermore, the two higher doses (14 and 42 ppm) significantly increased the lifespan of male mice, including both maximum and median lifespan extension (Miller et al., 2014).

The impact of administering rapamycin to male and female mice after birth was also reported. Specifically, rapamycin at a dosage of 10 mg/kg was given to the mice from postnatal day 4 to day 30 (P4–P30) or from day 30 to day 60 (P30–P60). In the P4–P30 group, both genders exhibited an approximate 9.6% increase in maximum lifespan, whereas no notable alterations were observed in the P30–P60 group (Aiello et al., 2022).

#### 3.3.5 Effects of rapamycin on human health

While its beneficial effects on lifespan extension are well‐documented in model organisms, the application to human aging remains exploratory. Notably, a recent study on the mTOR inhibitor RAD001 offers promising insights, showing improved immune function in elderly humans, as evidenced by enhanced responses to influenza vaccination. This finding opens the door to further investigations into whether mTOR inhibition can positively impact a broader range of aging‐related conditions in humans (Mannick et al., 2014, 2018).

In humans, it is worth nothing that although people on an “every other day” diet with resveratrol seemed to live longer, this difference was not statistically significant (Pearson et al., 2008). Another study conducted in overweight and slightly obese patients for 4 weeks did not confirm any significant differences in the cardiovascular and metabolic risk markers between groups, after daily resveratrol intake. Presently, several clinical trials are being conducted to assess the safety and effectiveness of resveratrol in humans, and initial findings have shown promise (Berman et al., 2017; de Cabo et al., 2014; Kawamura et al., 2020; Longo et al., 2015; Ramírez‐Garza et al., 2018).

Increased oxidative stress has been associated with muscular dystrophy. Resveratrol, an activator of the protein deacetylase SIRT1 and an antioxidant, decreases muscular and cardiac oxidative damage and improves pathophysiological conditions in animal models of muscular dystrophy (MD). This suggests that resveratrol may offer therapeutic benefits to patients with muscular dystrophies (Kawamura et al., 2020).

Other phase I, II, and III clinical trials have utilized rapamycin to inhibit the signal transduction induced by most cytokines, offering a unique method to both disengage the immune response and inhibit injuries not dependent on antigens (Taner et al., 2005).

Unfortunately, all studies involving humans were conducted on patients suffering from various diseases.

### 3.4 Spermidine

Spermidine is a naturally occurring polyamine that is present in a variety of foods and is gaining recognition for its potential health benefits (Kiechl et al., 2018). Spermidine is involved in several cellular processes, including DNA synthesis, cell growth, and autophagy. Spermidine has been shown to possess anti‐inflammatory and antioxidant properties and has been the subject of several studies for its potential role in the aging process and age‐related diseases. While the full extent of its benefits has yet to be understood, spermidine is gaining attention as a promising agent for promoting health and wellness, especially as it relates to aging. With ongoing research, we may discover more about the potential benefits of spermidine and how it can be used to improve health and longevity.

#### 3.4.1 Effects of spermidine on the lifespan of *Saccharomyces cerevisiae*

The anti‐aging effects of spermidine have been initially tested on yeast cells as a model system. Chronological aging experiments revealed that yeast cells lacking ornithine decarboxylase experienced increased mortality and a shortened lifespan. However, supplementation with spermidine, particularly at low doses of 0.1 mM, led to a remarkable increase in lifespan, up to fourfold compared to untreated cells. Additionally, higher concentrations of spermidine were found to extend the lifespan of wild‐type yeast cells with various genetic backgrounds. The specific doses of spermidine used in this study demonstrated its significant impact on longevity in yeast. Furthermore, spermidine treatment not only slowed down chronological aging but also rejuvenated replicative old yeast cells. These long‐lived yeast cells also exhibited improved stress resistance. These findings underscore the potential of spermidine in promoting cellular longevity, with low doses showing substantial benefits in terms of lifespan extension (Eisenberg et al., 2009). A more recent study confirmed previous results, although the median survival was extended only by 33% (Su et al., 2021).

#### 3.4.2 Effects of spermidine on the lifespan of *Drosophila melanogaster*

A study conducted by Eisenberg et al. explored the extension of longevity through the stimulation of autophagy and examined the mechanisms involved. The study found that supplementing the *DM* W1118 strain with varying concentrations of spermidine (0.01, 0.1, 1, 4, and 10 mM) for both male and female flies resulted in an increased lifespan of up to 30%, particularly at the concentration of 1 mM (Eisenberg et al., 2009).

#### 3.4.3 Effect of spermidine on the lifespan of *Caenorhabditis elegans*

One study found that treating *C. elegans* with 0.2 mM spermidine upon reaching adulthood led to an increased lifespan of up to 15%. Next, the investigators tested the hypothesis that spermidine extends the lifespan by modulating autophagy. They compared the lifespan of wild‐type nematodes with that of the autophagy‐deficient *bec‐1* (*RNAi*) strain and found that the MLS of the wild‐type strain was about 25% higher than that of the *bec‐1* (*RNAi*) strain (Eisenberg et al., 2009).

The experiments conducted in the same study provided support for the positive effects of spermidine on the lifespan of *C. elegans*. Both the wrn‐1 (gk99) and N2 wild‐type strains were treated with varying concentrations of spermidine (1, 5, 10, and 20 mM). The most effective dose was found to be 5 mM, which resulted in a lifespan extension of approximately 36%.

#### 3.4.4 Effects of spermidine on the lifespan of mice

A study was conducted to examine the effect of polyamine administration on cardiovascular diseases in both male and female C57BL6 mice. The study divided the mice into two groups: one receiving lifelong treatment (starting at 4 months) and the other receiving late‐in‐life treatment (starting at 18 months). The results showed that the lifespan of the mice increased by approximately 10% under polyamine administration. However, it was observed that the group receiving lifelong treatment had a higher likelihood of experiencing toxic effects compared to the group receiving late‐in‐life treatment (Eisenberg et al., 2016).

Finally, administration of spermidine enhanced cardiac autophagy, mitophagy, and mitochondrial respiration. Additionally, it improved the mechanical and elastic properties of cardiomyocytes in vivo, accompanied by elevated titin phosphorylation and the suppression of subclinical inflammation. However, in mice deficient in the autophagy‐related protein Atg5 specifically within cardiomyocytes, spermidine supplementation did not convincingly confer cardioprotective effects. This suggests that the primary beneficial effect of spermidine is to enhance autophagy in cardiomyocytes (Eisenberg et al., 2016).

Despite numerous studies demonstrating an increase in lifespan in lower and higher organisms such as mice, our research team did not observe any statistically significant outcomes in a study involving Sprague–Dawley male rats treated with a daily dosage of 25 mg/kg of spermidine, starting from 18 months of age. However, we did observe notable enhancements in behavioral tests assessing motor function, anxiety, and memory. Additionally, when examining brain tissue, we observed an improvement in brain autophagy and a reduction in neuroinflammation (Filfan et al., 2020).

#### 3.4.5 Effects of spermidine on human health

A recent study examined the effect of spermidine on peripheral blood mononuclear cells isolated from young and healthy individuals (under 35 years old). The cells were treated in vitro with different concentrations of spermidine (0 nM, 0.2 nM, 2 nM, 20 nM, and 2 μM) for 12 days. The authors investigated the effects of spermidine supplementation by comparing the survival rates of cells treated at 1 and 7 days after extraction. The results showed that the control group had a survival rate of approximately 15%. In contrast, the cells supplemented with spermidine at a 20 nM dose demonstrated a survival rate of up to 50% (Eisenberg et al., 2009).

Recently, a small population‐based study has shown that nutrition rich in spermidine, divided into three groups (<62.2 μmol/day; 62.2–79.8 μmol/day; >79.8 μmol/day) based on quinquennial food‐frequency questionnaires is linked to increased survival in humans. The survival advantage was driven by a reduced risk of death from all major causes (Kiechl et al., 2018).

In a preclinical research study, a 28‐day toxicity test was conducted on a group of mice using the highest dose of 60 mg/kg of spermidine. Post‐mortem histological data revealed no significant organ damage, except for a 12% increase in kidney weight compared to the control group. Subsequently, the authors performed a translational study to examine the impact of spermidine supplementation using a spermidine‐rich plant extract equivalent to 41 mg/kg on cognitive function, mental status, and laboratory parameters in humans. The reported results showed no significant differences in weight, vital signs, clinical chemistry, and hematological parameters of safety. Additionally, there were no discernible differences in self‐reported physical and mental health between the spermidine and placebo‐treated groups at the end of the 3‐month intervention. The spermidine treatment was deemed safe and well‐tolerated (Schwarz et al., 2018).

### 3.5 Chloroquine

Chloroquine (CQ), known for its effectiveness in treating malaria and reducing inflammation, has been employed for various medical conditions including systemic lupus erythematosus and rheumatoid arthritis. Recently, CQ has also shown potential in the treatment of cancers and viral infections (Zhou et al., 2020).

#### 3.5.1 Effects of chloroquine on the lifespan of mice

The role of CQ as an autophagy inhibitor has garnered recent attention, with investigations focusing on its potential opposing effect to spermidine on lifespan in model organisms (Doeppner et al., 2022). Against all odds, male NMRI mice treated with chloroquine at a dose of 50 mg/kg/day, starting at 500 days old and continuing for 286 days, significantly outlived the control group, with an average lifespan of 786 days compared to 689 days in the controls. Furthermore, the median lifespan and the maximum lifespan were also significantly different between the two groups. Chloroquine treatment at a dose of 50 mg/kg extended the median lifespan of middle‐aged NMRI male mice by 11.8% and the maximum lifespan by 11.4% (Doeppner et al., 2022).

#### 3.5.2 Effect of chloroquine on the lifespan of rats

Another study treated 24‐month‐old Sprague–Dawley rats with a low dose of chloroquine (0.1 mg/kg) twice a week for 5 months. The study demonstrated an extended lifespan in aged rodents, with a 6% increase in median lifespan and a 13% increase in maximum lifespan. This effect was attributed to the reduction of tissue fibrosis and chronic inflammation (Li et al., 2022).

## 4 DISCUSSION

In this narrative review, we explored the translatability of findings from longevity studies utilizing diverse drug treatments across various species and their potential applications to humans. It is important to note that our study specifically excluded nutritional approaches, such as caloric restriction and intermittent fasting. The results are summarized in Tables S1–S5.

While our study primarily focuses on the effects of non‐genetic (pharmacological) interventions on lifespan across various species, it is important to acknowledge the significant contributions of genetic studies to our understanding of aging. Notably, a wide‐scale comparative analysis of longevity genes and interventions has revealed that manipulations of orthologous longevity genes often result in concordant effects on lifespan across different model organisms, despite their significant evolutionary distance. This underscores the potential universality of certain aging mechanisms and highlights the importance of both genetic and pharmacological approaches in unraveling the complexities of aging. Such findings complement our pharmacological intervention data by suggesting that targeting fundamental biological pathways, whether through genetic or pharmacological means, holds promise for influencing lifespan and healthspan across species (Yanai et al., 2017).

The pursuit of increasing healthy lifespan is of interest to many academic disciplines, including biogerontology, medicine, and biochemistry (Harrison et al., 2009). In recent years, numerous studies have investigated the potential of diverse treatments in prolonging life and delaying age‐related illnesses in animal models. Well‐documented treatments across species of increasing complexity include drugs such as rapamycin, resveratrol, spermidine, chloroquine, and even medications historically employed for treating different diseases, like metformin, which is used in the management of type 2 diabetes (Cabreiro et al., 2013; Martin‐Montalvo et al., 2013).

**FIGURE 1:**
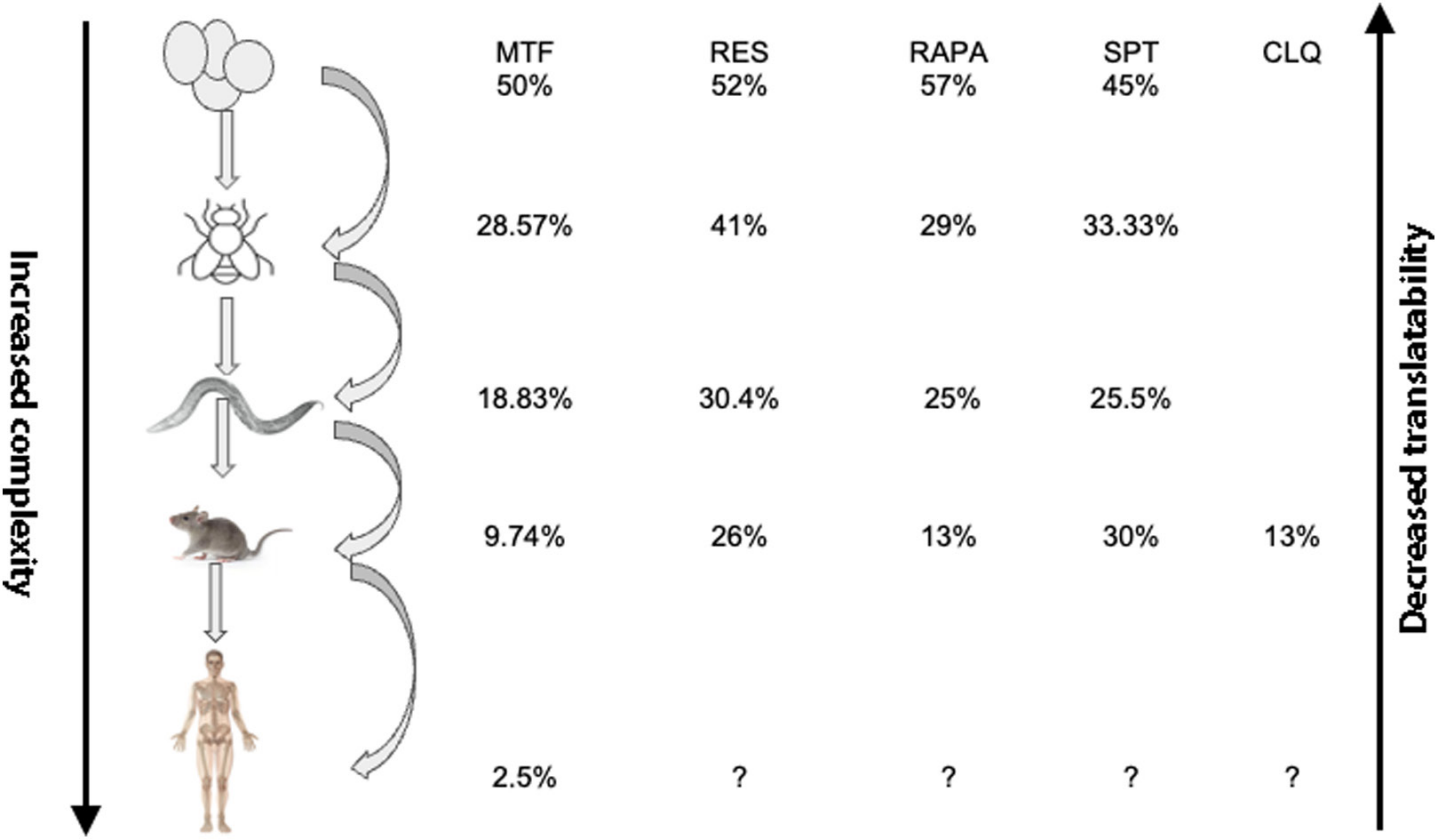
The decreasing magnitude of the positive effect with increasing complexity in anti‐aging treatments is obvious. For example, we noted that the positive effects of metformin decreased from 50% in *Saccharomyces cerevisiae* to negligible (if any) effects in humans. Similarly, the effects of resveratrol decreased almost linearly from 70% in *S. cerevisiae* to 41% in *DM*, to 30% in *Caenorhabditis elegans*, to 26% in rodents. In humans, it is worth noting that although people on an “every other day” diet with resveratrol seemed to live longer, this difference was not statistically significant. For details, see Tables S1–S5.

The decreasing magnitude of the positive effect with increasing complexity in anti‐aging treatments is obvious (Figure 1). Thus, we noted the positive effects of metformin decreased from 50% in *S. cerevisiae* to negligible (if any) effects in humans. Similarly, the effects of resveratrol decreased almost linearly from 70% in *S. cerevisiae* to 41% in *DM*, to 30% in *C. elegans*, to 26% in rodents.

The impact of rapamycin on lifespan across species decreased progressively, from 57% in *S. cerevisiae* to approximately 29% in *DM*, further declining to 25% in *C. elegans*, and ultimately reaching 13% in rodents (Figure 1).

Among the interventions explored, rapamycin, through its inhibition of the mTOR pathway, has been documented to extend lifespan and delay aging‐associated diseases across numerous species, signifying its role in regulating aging processes (Johnson et al., 2013). Various mechanisms have been proposed to explain the effect of rapamycin on longevity. Specifically, the regulation of the mTORC1 component raptor (*daf‐15*) by rapamycin has been demonstrated to extend lifespan in the nematode *C. elegans*. Further studies conducted on the fruit fly *D. melanogaster* have shown that mutations in mTOR and various other components of the mTORC1 pathway can also prolong lifespan in these organisms (Johnson et al., 2013; Kaeberlein et al., 2015; Sharp & Strong, 2023). Decreased signaling via the insulin/IGF‐1 pathway (IIS) has been linked to increased lifespan in species such as nematodes, fruit flies, and mice. The activity of mTOR is stimulated by IIS through the action of AKT, and mTORC1 has the ability to downregulate IIS via S6K, which in turn suppresses the insulin receptor substrate 1 (IRS‐1). Further research has shown that in mammalian cells, FOXO3A can control the expression of tuberous sclerosis protein 1 (TSC1), a regulatory factor that inhibits mTORC1. Additionally, it has been found that 4E‐BP1, a target of mTORC1, is also regulated by FOXO in fruit flies (Johnson et al., 2013; Mannick & Lamming, 2023).

It has been proposed that aging and age‐associated illnesses are influenced by nutrient detection. Thus, it is hypothesized that the overall nutritional status within multicellular organisms is monitored via both overarching and cellular‐level nutrient detection mechanisms. The overarching system governs the release of hormonal signals into the bloodstream, such as GH, IGF‐1, and insulin. These hormones interact with cell surface receptors, triggering pathways inside the cell related to nutrient signaling. On a cellular level, indicators like ATP/ADP ratios, NADH/NAD+ levels, amino acid concentrations, and the status of ribosome assembly directly influence cellular pathways, thereby altering how cells respond to hormones in the bloodstream. The way in which the various tissues and cell types of multicellular organisms respond is intricately managed through the unique expression of cellular factors and receptors, the specific nature of systemic factors, and the presence of tissue‐specific and circulating elements that adjust biological activity. These complex networks of signals are designed to prioritize growth and reproduction over cellular repair and maintenance. Persistent activation of these nutrient‐sensing signals is a key factor in the process of aging and the development of various chronic conditions that often accompany older age (Johnson, 2018).

Thus, now we know that if any anti‐aging rapamycin treatment is to be effective, it should commence after growth is completed. This timing is crucial to directly inhibit aging without affecting developmental growth. Of note, in post‐development, mTOR is hyperfunctional and, therefore, a suitable anti‐aging target for inhibition (Blagosklonny, 2022).

The anti‐aging drug spermidine is relatively new. The autophagy‐inducing agent, spermidine, has become available, which may mirror the effects of caloric restriction. Autophagy plays a vital role in cellular balance, and its dysregulation can lead to various issues including impaired stress responses. Although it did show positive results for lifespan extension in lower organisms, a recent study on aged rats showed that spermidine, when given in drinking water, did not extend either the median or the maximum lifespan of middle‐aged male Sprague–Dawley rats. However, spermidine treatment had a beneficial effect on body weight and was associated with a reduction in anxiety and an increase in curiosity, as assessed by exploratory behavior.

Other positive effects of spermidine treatment were recently reported on mice fertility. Mice give birth to litters, and their litter size is affected by aging. Young mice often give birth to 14 pups at a time, while older mice normally give birth to only three. However, older mice given spermidine gave birth to an average of six (Zhang et al., 2023).

In humans, longer‐term spermidine supplementation in participants with subjective cognitive decline did not modify memory and biomarkers compared to the placebo. However, exploratory analyses indicated possible beneficial effects on verbal memory and inflammation, which need validation in future studies at higher dosages (Schwarz et al., 2022).

Notably, antidepressants influence autophagy pathways. Spermidine supplementation might offer benefits for psychiatric conditions, presenting a safe, affordable, and easy adjunct to current treatments. The project's goal is to study the clinical effects of spermidine, correlated with molecular profiling (ClinicalTrials.gov ID NCT04823806).

A new anti‐aging agent is chloroquine, the effect of which was discovered on mice longevity during the COVID pandemic (Doeppner et al., 2022). Several studies are underway in our laboratory to investigate the effect of chloroquine on lifespan and health of mice and rats.

## 5 CONCLUSIONS

Although animal studies offer valuable insights into biological mechanisms and potential interventions, their applicability to species of varying complexity, including humans, has been limited. Despite of these difficulties, there is an increasing curiosity in investigating the possibilities of longevity interventions in the human population.

The translatability of longevity treatments to humans can be influenced by various factors, including the dosage or schedule employed in animal studies. There are instances where the dosages utilized in animal studies may not be practical or safe for human use, necessitating adjustments to achieve the intended outcome. Furthermore, variances in diet, gender, and strain among animal models can also impact the efficacy of treatments, posing difficulties in extrapolating the findings to humans (Schwarz et al., 2018; van der Made et al., 2015).

The limited translatability between species of increasing complexity can be explained by a number of factors:

1. The effectiveness or significance of the targeted molecule from a pathway might differ in various metabolic scenarios. For example, the anti‐aging mechanisms of resveratrol primarily involve ameliorating oxidative stress by scavenging ROS. However, ROS play a more significant role in flying species like Drosophila than in mammals, which may possess additional mechanisms to counteract ROS. Indeed, recent research has revealed a more complex and beneficial role of ROS in regulating metabolism, development, and lifespan (Popa‐Wagner et al., 2013; Santos et al., 2018).
2. Second, the weight of targeted signaling pathways differs for a species' general metabolism. Therefore, single mutations that reduce insulin/IGF‐1 signaling can significantly increase the lifespan of simple organisms such as *C. elegans* and *D. melanogaster*. However, the increased complexity of the pathway, attributed to additional regulators like insulin and growth hormone, has made it challenging to distinguish the roles of each key component in mammalian longevity.
3. Third, redundancy in pathways is a widespread phenomenon in species of increasing complexity, observed across all forms of life. It has developed as a safeguard against disturbances that might otherwise interfere with essential processes, such as mutations or shifts in the environment. Thus, blocking one pathway does not necessarily impede the cellular or organismal process.
4. Forth, as we make progress into research on anti‐aging therapies, the challenges posed by the increasing complexity of species remind us that, much like many aspects of biology and medicine, there exists a law of diminishing returns. While the initial interventions may yield significant and noticeable impacts, the subsequent benefits might be less pronounced with the addition of more layers of complexity and control.

Additional research is necessary to assess the safety and efficacy of these findings within human populations. Various factors, such as genetic variations, metabolic disparities, the age at which treatment begins, duration of treatment, dosage, and environmental influences, can all affect the effectiveness of treatments across different species. For instance, certain treatments that extend lifespan in rodents may not yield identical outcomes in humans due to differences in drug metabolism or pharmacokinetics. We believe that mice genetics, enabling to pinpoint crucial pathways involved in longevity and medical genetics offering drugs developed using gene technologies on are the key for future studies on aging.

The future of aging research, especially in mouse and medical genetics for drug development using gene technologies, is very promising. Mouse genetics provides deep insights into aging mechanisms, potentially leading to human aging process breakthroughs. Medical genetics offers prospects for targeted therapies that could slow aging or lessen its negative effects, with gene technology enabling personalized medicine for more effective treatments with fewer side effects. Additionally, combining computational models, bioinformatics, and systems biology could fast‐track the identification of genes and pathways related to longevity, improving our grasp of factors that influence lifespan and healthspan. In the future, prospects for targeted therapies could benefit from two comprehensive databases on anti‐aging drugs and interventions, DrugAge (Barardo et al., 2017) and Geroprotectors (Moskalev et al., 2015).

Finally, with the increasing complexity of organisms, targeting only one pathway is unlikely to consistently extend lifespan. Healthy aging might be a more realistic aim of anti‐aging research.

## AUTHOR CONTRIBUTIONS

Conceptualization, A.P.‐W. and C.C.; methodology and investigation, D.B; writing—original draft preparation, A.P.‐W. and D.B.; review and editing, D.H., T.D. and A.G.; funding acquisition, A.P.‐W. and D.H.

## FUNDING INFORMATION

This work was supported by “The National Recovery and Resilience Plan” set up by the European Union “Targeting macrophages/monocytes in the aged ischemic brain by pharmacological, genetic and cell‐based tools” (project 760058; to DH), Executive Agency for Higher Education, Research, Development and Innovation Funding (UEFISCDI) (project PN‐III‐P4‐ID‐PCE‐2020‐059; to AP‐W), German Research Foundation (DFG; project 514990328; to DH) and German Federal Ministry of Education and Science (BMBF; project 161L0278B (3DOS); to DH und TRD).

## CONFLICT OF INTEREST STATEMENT

No conflicts of statement to declare.

## Supporting information


Tables S1–S5

## Data Availability

The data that support the findings of this study are available from the corresponding author upon reasonable request.
