# Supplementary material for: Translatability of life‐extending pharmacological treatments between different species
Source: Aging Cell. 2024 May 26;23(7):e14208. doi: 10.1111/acel.14208 (PMC11258477; doi:10.1111/acel.14208)
Supplement: Supplementary file 1 — Tables S1–S5 [file ACEL-23-e14208-s001.docx]

**Table S1. The translatability of the life-extending treatment effect on gender, diet, and treatment doses**

| Reference | Treatment | Species | Is the effect dependent on the gender? | Is the effect dependent on diet? | Is the effect dependent on the dose? |
| --- | --- | --- | --- | --- | --- |
| Şeylan, 2003 | MTF | *S. pombe* | - | Y | - |
| Avelar-Rivas, 2020 | MTF | *S. cerevisiae* | - | NS | - |
| Abrat, 2018 | MTF | *D. melanogaster* | Y | Y | Y |
| Kokott-Vuong, 2021 | MTF | *D. melanogaster* | NS | NS | NS |
| Slack, 2012 | MTF | *D. melanogaster* | Y | NS | Y |
| Cabreiro, 2013 | MTF | *C. elegans* | - | Y | Y |
| Espada, 2020 | MTF | *C. elegans* | - | N | Y |
| Martin-Montalvo, 2013 | MTF | Mice | - | - | Y |
| Arkadiev, 2011 | MTF | Mice | NS | NS | NS |
| Stevenson-Hoare, 2023 | MTF | Humans | NS | NS | NS |
| Howitz, 2003 | RES | *S. cerevisiae* | - | - | Y |
| Jarolim, 2004 | RES | *S. cerevisiae* | - | Y | Y |
| Pannakal, 2017 | RES | *S. cerevisiae* | - | - | Y |
| Wang, 2013 | RES | *D. melanogaster* | Y | Y | Y |
| Islam, 2019 | RES | *D. melanogaster* | N | Y | Y |
| Khan, 2019 | RES | *D. melanogaster* | Y | Y | N |
| Bauer, 2004 | RES | *D. melanogaster* | Y | Y | Y |
| Lee, 2016 | RES | *C. elegans* | - | NS | Y |
| Rea, 2005 | RES | *C. elegans* | - | NS | y |
| Gruber, 2007 | RES | *C. elegans* | - | NS | Y |
| Fischer, 2017 | RES | *C. elegans* | - | NS | N |
| Baur, 2006 | RES | Mice | - | Y | Y |
| Miller, 2011 | RES | Mice | Y | Y | Y |
| Alvers, 2009 | RAPA | *S. cerevisiae* | - | - | Y |
| Rallis, 2013 | RAPA | *S. pombe* | - | Y | Y |
| Aiello, 2022 | RAPA | *D. melanogaster* | Y | NS | Y |
| Schinaman, 2019 | RAPA | *D. melanogaster* | NS | - | - |
| Bjedov, 2010 | RAPA | *D. melanogaster* | Y | Y | Y |
| Villa-Cuesta, 2014 | RAPA | *D. melanogaster* | NS | Y | Y |
| Yee, 2021 | RAPA | *C. elegans* | - | NS | NS |
| Robida- Stubbs, 2012 | RAPA | *C. elegans* | - | NS | Y |
| Harrison, 2009 | RAPA | Mice | Y | Y | - |
| Miller, 2011 | RAPA | Mice | Y | - | - |
| Miller, 2014 | RAPA | Mice | Y | - | Y |
| Aiello, 2022 | RAPA | Mice | Y | - | - |
| Doeppner, 2022 | CLQ | Mice | - | - | - |
| Li, 2022 | CLQ | Rat | - | - | - |
| Eisenberg, 2009 | SPT | *S. cerevisiae* | - | - | Y |
| Su, 2021 | SPT | *S. cerevisiae* | - | - | - |
| Eisenberg, 2009 | SPT | *D. melanogaster* | NS | - | Y |
| Eisenberg, 2009 | SPT | *C. elegans* | - | - | - |
| Yang, 2020 | SPT | *C. elegans* | - | - | Y |
| Eisenberg, 2009 | SPT | Human peripheral blood mononuclear cells | NS | - | Y |
| Eisenberg, 2016 | SPT | Mice | NS | - | - |
| Filfan, 2020 | SPT | Rat | - | - | - |
| Kiechl, 2018 | SPT | Humans | - | Y | Y |
| Schwarz., 2018 | SPT | Humans | - | - | - |

*Abbreviations*: Y- Yes; N- No; NS- Not specified/not investigated

**Table S2. Translatability of life-extending treatment with metformin between different species of increasing complexity**

|  | **Species** | | | | |
| --- | --- | --- | --- | --- | --- |
| **Species x Species** | *S cerevisiae* | *D. melanogaster* | *C elegans* | Mice | Humans |
| *S cerevisiae* | 100% | 57.14% | 37.66% | 19.48% | 5% |
| *D. melanogaster* | 175% | 100% | 65.9% | 34.09% | 8.75% |
| *C. elegans* | 265% | 151.72% | 100% | 51.72% | 13.27% |
| Mice | 513.34% | 293.32% | 193.32% | 100% | 25.66% |
| Humans | 2000% | 1142.8% | 753.2% | 389.6% | 100% |

**Table S3. Translatability of life-extending treatment with resveratrol between different species**

**of increasing complexity**

|  | **Species** | | | | |
| --- | --- | --- | --- | --- | --- |
| **Species x Species** | *S cerevisiae* | *D. melanogaster* | *C elegans* | Mice | Humans |
| *S cerevisiae* | 100% | 78.84% | 58.46% | 50% | - |
| *D. melanogaster* | 126.82% | 100% | 74.14% | 63.41% | - |
| *C. elegans* | 171.05% | 134.86% | 100% | 85.52% | - |
| Mice | 200% | 157.69% | 116.92% | 100% | - |
| Humans | - | - | - | - | - |

**Table S4. Translatability of life-extending treatment with rapamycin between different species**

**of increasing complexity**

|  | **Species** | | | | |
| --- | --- | --- | --- | --- | --- |
| **Species x Species** | *S cerevisiae* | *D. melanogaster* | *C elegans* | Mice | Humans |
| *S cerevisiae* | 100% | 50.87% | 43.85% | 22.80% | - |
| *D. melanogaster* | 196.55% | 100% | 86.20% | 44.82% | - |
| *C. elegans* | 228% | 116% | 100% | 52% | - |
| Mice | 438.46% | 226.07% | 192.30% | 100% | - |
| Humans | - | - | - | - | - |

**Table S5. Translatability of life-extending treatment with spermidin between different species of increasing complexity**

|  | **Species** | | | | |
| --- | --- | --- | --- | --- | --- |
| **Species x Species** | *S cerevisiae* | *D. melanogaster* | *C elegans* | Mice | Humans |
| *S cerevisiae* | 100% | 74.06% | 56.6% | 28.8% | - |
| *D. melanogaster* | 135.135% | 100% | 76.5% | 90.09% | - |
| *C. elegans* | 176.47% | 130.7% | 100% | 117.6% | - |
| Mice | 150% | 111.1% | 85% | 100% | - |
| Humans | - | - | - | - | - |
